# Supplementary material for: Breakpoint modelling of temporal associations between non-pharmaceutical interventions and symptomatic COVID-19 incidence in the Republic of Ireland
Source: PLoS One. 2021 Jul 29;16(7):e0255254. doi: 10.1371/journal.pone.0255254 (PMC8321012; doi:10.1371/journal.pone.0255254)
Supplement: S1 Table — (DOCX) [file pone.0255254.s001.docx]

| S1 Table. Pobal HP deprivation index components and descriptions | | |
| --- | --- | --- |
| Component Label | **Component Name** | **Component Description** |
|  |  |  |
| HPabs | Absolute HP Index Score | Composite measure of deprivation calculated for each SA, measured on a single scale across all census periods |
| HPrel | Relative HP Index Score | A measure of the level of deprivation in each SA relative to all other small areas surveyed |
| TOTPOP | Total population | Total population in each SA during each census period |
| POPCHG | Population Change | Percentage increase in population over the previous five years |
| AGEDEP | Age dependency rate | Percentage of population aged under 15 or over 64 years of age |
| LONEPA | Lone parent ratio | Percentage of households with children aged under 15 years and headed by a single parent |
| EDLOW | Primary education | Percentage of people in each SA with primary education as their highest level of education attainment |
| EDHIGH | Third level education | Percentage of people in each SA with third level education as their highest level of education attainment |
| HLPROF | Higher and lower professionals | Percentage of households headed by professionals or managerial and technical employees, including farmers with 100 acres or more |
| LSKILL | Proportion of semi-skilled and unskilled manual workers | Percentage of households in each SA headed by semi‐skilled or unskilled manual workers, including farmers with less than 30 acres |
| UNEMPM | Male unemployment rate | Rate of male unemployment in each SA |
| UNEMPF | Female unemployment rate | Rate of female unemployment in each SA |
| PEROOM | Persons per room | Mean number of persons per household room in each small area |
| LARENT | Local authority housing | Percentage of local authority housing in each SA |
| PRRENT | Privately rented housing | Percentage of privately rented housing in each SA |
| OHOUSE | Own home | Percentage of privately owned housing in each SA |
