# Supplementary material for: Breakpoint modelling of temporal associations between non-pharmaceutical interventions and symptomatic COVID-19 incidence in the Republic of Ireland
Source: PLoS One. 2021 Jul 29;16(7):e0255254. doi: 10.1371/journal.pone.0255254 (PMC8321012; doi:10.1371/journal.pone.0255254)
Supplement: S2 Table — (DOCX) [file pone.0255254.s002.docx]

| **S2 Table. Pobal HP Index absolute and relative deprivation index score classification** | |
| --- | --- |
| **Index Score** | **Level of deprivation/affluence** |
| **-40 to - 30** | Extremely disadvantaged |
| **-30 to -20** | Very disadvantaged |
| **-20 to -10** | Disadvantaged |
| **-10 to 0** | Marginally below average |
| **0 to 10** | Marginally above average |
| **10 to 20** | Affluent |
| **20 to 30** | Very affluent |
| **30 to 40** | Extremely affluent |

*Note.* Reprinted from the 2016 Pobal HP Deprivation Index for Small Areas

(SA): Introduction and Reference Tables, Trutz Haase Jonathan Pratschke,

September 2017
