# Supplementary material for: Breakpoint modelling of temporal associations between non-pharmaceutical interventions and symptomatic COVID-19 incidence in the Republic of Ireland
Source: PLoS One. 2021 Jul 29;16(7):e0255254. doi: 10.1371/journal.pone.0255254 (PMC8321012; doi:10.1371/journal.pone.0255254)
Supplement: Attachment — Submitted filename: ResponsetoReviewers_Boudou.docx [file pone.0255254.s003.docx]

PONE-D-21-10957
Breakpoint modelling of temporal associations between non-pharmaceutical interventions and the incidence of symptomatic COVID-19 in the Republic of Ireland

Response to Editor

Editor/Journal Comment 1:

Please ensure that you refer to Figure 1 in your text as, if accepted, production will need this reference to link the reader to the figure.

**Author Response 1:**

Amended as requested
